# Penile metastasis from rectal adenocarcinoma: a case report

**DOI:** 10.1186/s13104-017-2901-5

**Published:** 2017-11-06

**Authors:** Boubacar Efared, Gabrielle Atsame Ebang, Soufiane Tahirou, Layla Tahiri, Ibrahim Sory Sidibé, Fatimazahra Erregad, Aboubakry Sow, Nawal Hammas, Moulay H. Farih, Laila Chbani, Hinde El Fatemi

**Affiliations:** 1 Department of Pathology, Hassan II University Hospital, Fès, Morocco; 2 Department of Radiology, Hassan II University Hospital, Fès, Morocco; 3 Department of Urology, Hassan II University Hospital, Fès, Morocco; 4 Laboratory of Biomedical and Translational Research, Faculty of Medicine and Pharmacology, Sidi Mohamed Ben Abdellah University, Fès, Morocco; 5 Faculty of Medicine and Pharmacology, Sidi Mohamed Ben Abdellah University, Fès, Morocco

**Keywords:** Penis, Metastasis, Adenocarcinoma, Pathology

## Abstract

**Background:**

Despite its rich vasculature, the penis is rarely involved by metastasis. Since the first description of penile metastasis in 1870, fewer than 500 cases have been reported in the literature. The pelvic organs are the main source of primary tumors that metastasize to the penis.

**Case presentation:**

We report a case of a 46-year-old Arabic man who presented with erectile dysfunction and painful induration of the penile root. Eight months ago, he had undergone abdomino-perineal resection for rectal adenocarcinoma after neo-adjuvant chemotherapy. The histological evaluation of the resected specimen disclosed a ypT3N0 tumor with a poor therapeutic response (around 5%). An adjuvant chemotherapy by XELOX (oxaliplatin plus capecitabine) regimen has been prescribed for the patient. The magnetic resonance imaging (MRI) showed tumoral infiltration of penile structures and a biopsy of the corpora cavernosa was performed. The histological examination disclosed a penile metastasis from the patient’s previous rectal adenocarcinoma. The patient is still alive and continues his adjuvant therapy.

**Conclusion:**

Penile secondary tumors are very rare and usually occur in patients with advanced tumor stages. A diagnosis of penile metastasis should be considered in patients with a history of malignancies who present with genitourinary symptoms. These patients have a dismal prognosis as they often die in the year after the diagnosis.

## Background

Despite its rich and interconnected vasculature, the penis is very rarely involved by metastasis [1, 2]. Since the first case reported by Eberth in 1870, to date at least 480 cases of penile secondary tumors have been reported in the English literature through single case reports or small series, with a largest series of 17 cases reported by Chaux et al. [2, 3]. The primary tumors that metastasize to the penis are mostly located in the pelvis, especially genitourinary tumors from the bladder and the prostate, followed by rectosigmoid tumors. Other primary sites include the lung, kidney, liver, bone, etc. [2–6]. Penile metastasis are mainly metachronous and they are diagnosed with variable intervals after the primary tumors. Metastasis to the penis is often a sign of an advanced stage of the primary tumor with a very poor prognosis as most of reported cases have died before 12 months after the diagnosis of the penile involvement [1, 2, 7].

We report herein, a case of a penile metastasis from a rectal adenocarcinoma in a 46-year-old patient, treated 8 months previously by surgery after neoadjuvant radio-chemotherapy.

## Case presentation

**Fig. 1:**
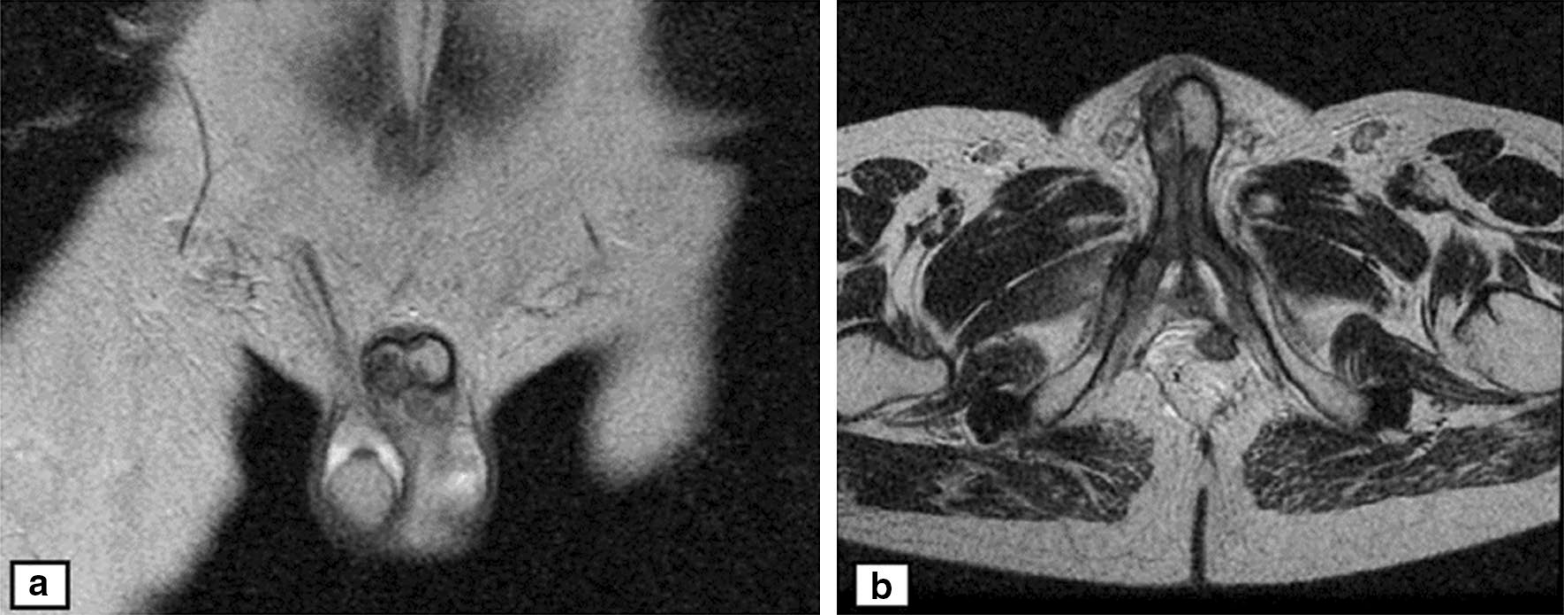
The magnetic resonance imaging (MRI) showing tumoral infiltration of the right corpora cavernosa (**a**), penile bulb and neighboring perineal soft tissue (**b**)

**Fig. 2:**
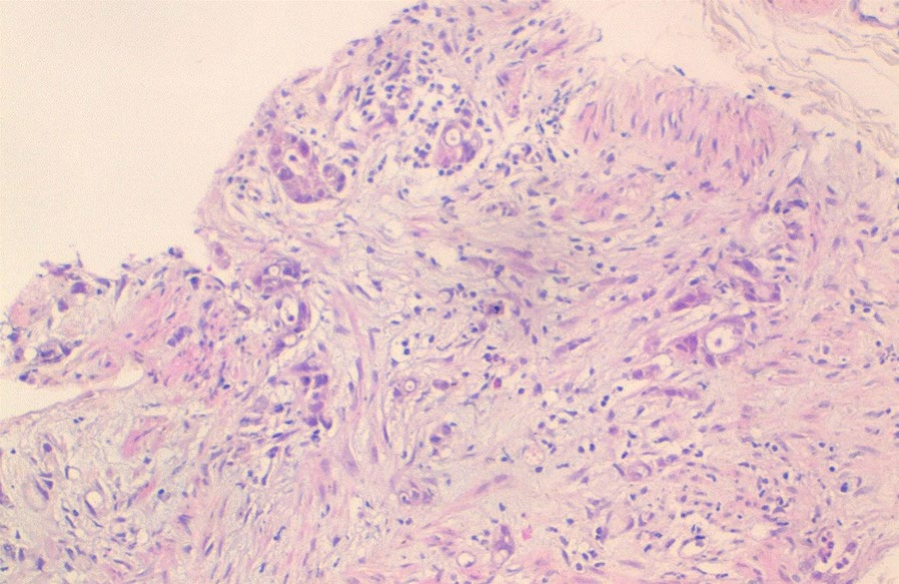
Tumoral glands invading the penile structures. Tumor cells had eosinophilic cytoplasm with oval nucleis with irregular contours (Hematoxylin–eosin-saffron ×200)

**Fig. 3:**
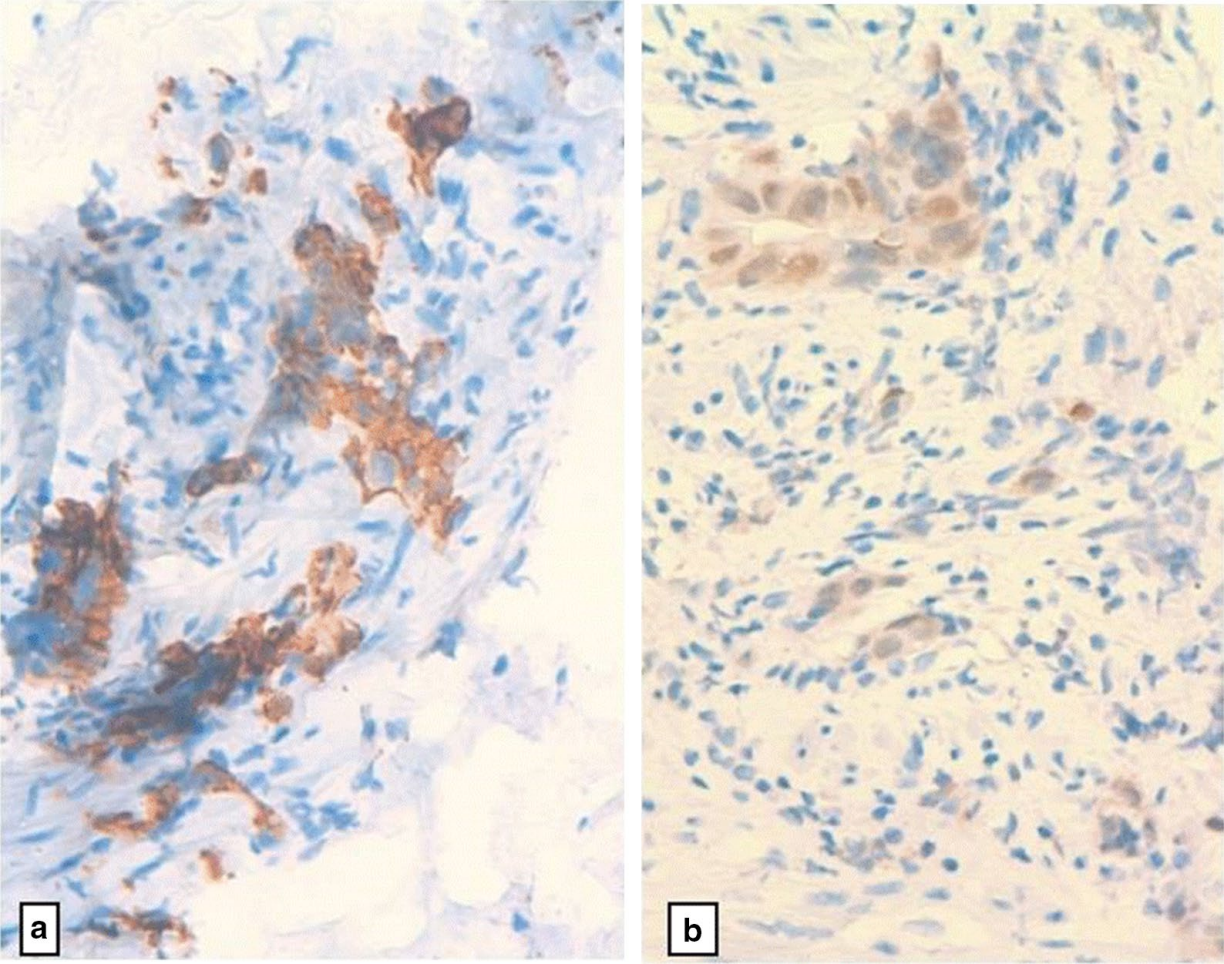
At immunohistochemistry, tumor cells were positive for cytokeratin 20 (CK20) (**a**) and CDX2 (Caudal type homeobox transcription factor 2) (**b**) (×400)

**Fig. 4:**
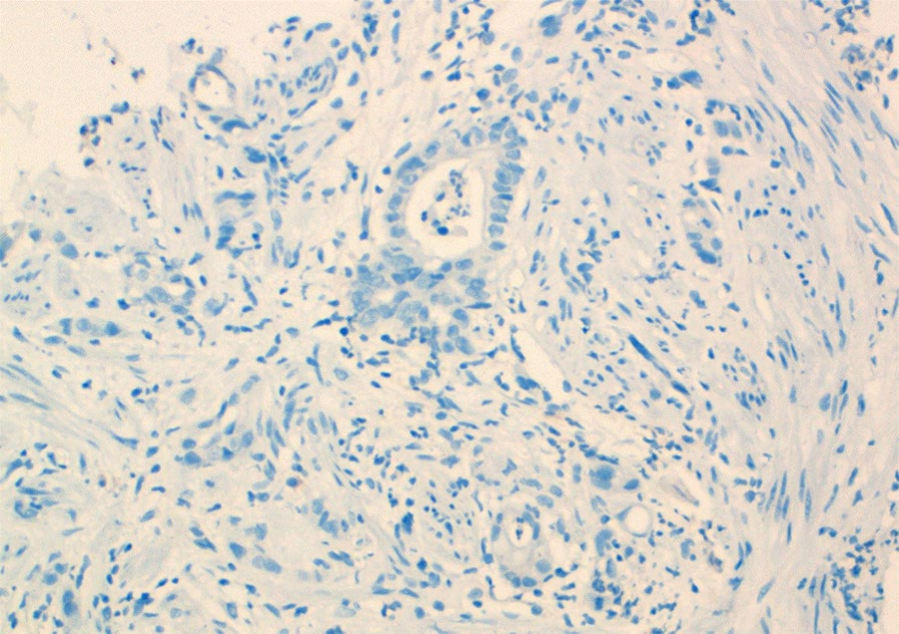
Tumor cells were negative for prostatic specific antigen (PSA) (Immunohistochemistry, × 400)

A 46-year-old Arabic man presented with a penile pain and erectile dysfunction for 6 months. Eight months previously, he had undergone abdomino-perineal resection for a moderately differentiated adenocarcinoma of the rectum. Before surgery, neo-adjuvant radio-chemotherapy had been prescribed for him. The pathological examination of his resected specimen disclosed a ypT3N0 tumor (American Joint Committee on Cancer (AJCC) 2009), with negative margins and a very poor therapeutic response (around 5%). There was no tumor instability, as tumor cells were positive for MLH1 (mutL homolog 1), MSH2 (mutS homolog 2), MSH6 (mutS homolog 6) and PMS2 (PostMeiotic segregation increased 2) at immunohistochemical evaluation. At multidisciplinary meeting (MDM), an adjuvant chemotherapy has been decided for the patient, with six cycles of XELOX regimen (capecitabine plus oxaliplatin). Eight months later, before the end of the adjuvant chemotherapy, he presented with a painful induration located at the right-lateral side of the penile root. The magnetic resonance imaging (MRI) showed tumoral infiltration of the right corpora cavernosa, penile bulb and neighboring perineal soft tissues (Fig. 1). A biopsy of the corpora cavernosa was performed and the histological examination on hematoxylin-eosin-saffron (HES) stained sections, showed tumoral glands invading the penile structures. Tumor cells had eosinophilic cytoplasm with oval nuclei and irregular contours (Fig. 2). At immunohistochemistry, tumor cells were positive for CK20 (cytokeratin 20) and CDX2 (caudal type homeobox transcription factor 2) (Fig. 3a, b), negative for CK7 (cytokeratin 7) and PSA (prostatic specific antigen) (Fig. 4). The diagnosis of penile metastasis from rectal adenocarcinoma has been disclosed. At present, the patient is still under his adjuvant chemotherapy (XELOX regimen).

## Discussion

Metastasis to the penis are very uncommon, and they are encountered in patients with advanced primary tumors. The vast majority of reported cases has presented with metachronous metastasis and had a history of known primary tumors [1–3, 8, 9]. The clinical presentation was usually an ulcerated or a hard mass located on the glans, the penis shaft or the penis root. Priapism, penis discharge, hematuria, pain, or urinary obstruction, have been reported as clinical symptoms in patients diagnosed with penile metastasis [1, 2]. In our case, the patient presented with a penile pain and erectile dysfunction for 6 months without any other clinical symptoms. The primary tumors that metastasize to the penis are widely from the genitourinary system accounting for approximately 70% of reported secondary tumors of the penis. Primary urinary bladder and prostatic tumors are the commonest metastatic tumors of this group (genitourinary system), followed by tumors from the kidney, testis, urethra, seminal vesicles, renal pelvis, and the ureter [2, 5, 6]. The gastrointestinal system is the second site of primary tumors that metastasize to the penis. In this group, colorectal primaries are the most reported tumors, other sites are very rarely encountered such as the pancreato-biliary system, the liver, the stomach, the esophagus, the tongue or the anal canal. Penile metastasis from the respiratory system (lung, upper airways), the bone, the skin, and other anatomical sites, are rarely reported compared to genitourinary and gastrointestinal systems that are commonly encountered in previously reported cases [1–6, 10].

Mostly penile metastasis present as metachronous tumors, however synchronous tumors have been reported [2, 3, 7]. The interval between the diagnosis of the primary tumor and the discovery of the penile secondary location varies from months to years (1 month–26 years) [3, 11]. Often, patients with penile metastasis presented with other organs involved by the secondary tumors [1, 3–5].

As patients presented usually with a known history of the primary tumors, any clinical symptoms involving the penis should prompt the search for an eventual penile secondary tumor. However, the clinical presentations are not specific and differential diagnosis have to be considered, such as penile primary malignancies (squamous cell carcinoma, melanoma, sarcoma), infectious diseases (syphilitic chancre, tuberculosis), non-tumoral cause of priapism, or Peyronie’s disease [3]. Several diagnostic imaging techniques can be used when a penile metastasis is suspected. The magnetic resonance imaging (MRI) is the best imaging tool as it allows a more accurate assessment of the tumor and its extent to the neighboring anatomic structures. The ultrasonography (US) or the computed tomography scan (CT-Scan) may have a valuable diagnostic utility but less than the MRI. The cavernosonography is an invasive technique that has no superior diagnostic value compared to non-invasive techniques (MRI, CT-Scan), and it is no longer used because of its important complications rate [1, 2]. A biopsy is needed for the histological confirmation of the penile metastasis. Often, metastatic tumors resemble their primaries, and a simple correlation with the patient’s history provides easily the correct diagnosis. Most penile metastatic tumors derive from prostatic adenocarcinomas, urinary bladder urothelial carcinomas, or adenocarcinomas from the gastrointestinal system [2, 11, 12]. A minimal immunohistochemical panel can prove useful in certain circumstances, for instance if the patient’s history is not known or if the histological features are not suggestive of any primary site. This panel can include antibodies against antigens commonly expressed by genitourinary or gastrointestinal tumors, such as cytokeratins (CKAE1/AE3, CK7, CK20, CK5/6), p63 (Tumor protein 63), PSA (prostatic specific antigen) or CDX2. In our case, even with the known history of the patient, the biopsy specimen is too small and we have used CK20, CK7, CDX2 and PSA, for an accurate diagnosis. Rare histologic types have been reported as penile secondary tumors, such as lung squamous carcinomas or adenocarcinomas, osteosarcoma, malignant melanoma, neuroendocrine tumors, sarcomas, cholangiocarcinoma etc. [3, 12, 13].

Despite its rich and interconnected vasculature, the penis is rarely involved by metastatic tumors. A number of theories have been postulated to explain the mechanisms by which primary tumor cells reach the penis. The retrograde venous route is thought to be the main way by which tumor cells from pelvic organs (prostate, urinary bladder, rectosigmoid) reach the corpus cavernosa and the glans, as the dorsal venous system of the penis has communications with the venous plexus system of the pelvis. Similarly, the retrograde lymphatic route seems to be the way by which tumor cells reach the penile skin via lymphatics that drain pelvic organs, passing through iliac and inguinal nodes. Less commonly, arterial spread, direct extension or iatrogenic spread by instrumentations, could explain metastasis from the lung and the liver primaries, sarcomas, or secondary penile root tumors from adjacent pelvic organs [1, 2]. In fact, our patient had corpus cavernosa, penile bulb and neighboring perineal soft tissues that were affected by the tumor. Direct extension or local recurrence could be discussed, but the patient had rectal adenocarcinoma classified as ypT3N0, meaning that the tumor was confined to the rectal subserosa with negative lymph nodes and negative margins.

The outcome of penile secondary tumors is very poor, as most of reported cases have died in the year following the diagnosis of the penile metastasis, with a median survival around 5 months [2, 3]. Penile metastasis as unusual tumors, little is known about them from pathophysiology to clinical management. Until now, there is no well designed and accepted management of patients with penile metastasis (penectomy or not?) leading unfortunately to a worse prognosis as patients die within months after the diagnosis. This unfortunate fact is likely due to insufficient data in the literature and there is an urgent need for more additional reported cases in order to improve the understanding of this rare entity, perhaps in the future effective management guidelines could be designed from consistent studies of all reported cases in the literature.

In our current case, the patient was relatively young (46 years) and a non-aggressive approach (chemotherapy) has been adopted and he is still alive with a stable disease. However, as reported previously in the literature, the management of penile metastasis is not clearly defined, and surgical penectomy does not seem to improve patients’ prognosis [3, 14, 15].

## Conclusion

Metastasis to the penis are very rare and occur mainly in patients with pelvic organs primary malignant tumors. Any clinical symptoms affecting the penile area in a patient with a history of a previous malignant tumor should prompt the search for an eventual secondary location. The prognosis of penile metastasis is very poor as they often reflect an advanced stage of the primary tumor.

## Abbreviations

MRI: magnetic resonance imaging
CT-scan: computed tomography scan
HES: hematoxylin–eosin-saffron
PSA: prostatic specific antigen
CKAE1/AE3: cytokeratin AE1/AE3 (pankeratin)
CK20: cytokeratin 20
CK7: cytokeratin 7
p63: tumor protein 63
CDX2: caudal type homeobox transcription factor 2
AJCC: American Joint Committee on Cancer
MSH2: mutS homolog 2
MSH6: mutS homolog 6
MLH1: mutL homolog 1
PMS2: PostMeiotic segregation increased 2
XELOX regimen: capecitabine plus oxaliplatin
